# Genome analysis of *Salmonella enterica* subsp. *diarizonae* isolates from invasive human infections reveals enrichment of virulence-related functions in lineage ST1256

**DOI:** 10.1186/s12864-018-5352-z

**Published:** 2019-01-31

**Authors:** Joaquín Giner-Lamia, Pablo Vinuesa, Laura Betancor, Claudia Silva, Julieta Bisio, Lorena Soleto, José A. Chabalgoity, José Luis Puente, The Salmonella CYTED Network, Fernando C. Soncini, Eleonora García-Vescovi, Griselda Flores, José Pedraza, Lucia Yim, Coralith García, Lizeth Astocondor, Theresa Ochoa, Noemí Hinostroza, M. Graciela Pucciarelli, Alfredo Hernández-Alvarez, Victor del Moral, Francisco García-del Portillo

**Affiliations:** 1 Laboratorio de Patógenos Bacterianos Intracelulares, Centro Nacional de Biotecnología-Consejo Superior de Investigaciones Científicas (CNB-CSIC), Madrid, Spain; 2 Centro de Biotecnología y Genómica de Plantas (CBGP), Universidad Politécnica de Madrid (UPM), Madrid, Spain; 3 Centro de Ciencias Genómicas, Universidad Nacional Autónoma de México, Cuernavaca, Morelos Mexico; 4 Instituto de Higiene, Facultad de Medicina, Universidad de la República, Montevideo, Uruguay; 5 Departamento de Microbiología Molecular, Instituto de Biotecnología, Universidad Nacional Autónoma de México, Cuernavaca, Morelos Mexico; 6 Ministerio de Salud de Bolivia, Centro Nacional de Enfermedades Tropicales (CENETROP), Santa Cruz, Bolivia; 7 Universidad Autónoma Gabriel René Moreno, Santa Cruz, Bolivia

**Keywords:** *Salmonella enterica*, Subspecies *diarizonae*, Invasive human infections, Comparative genomics, Virulence genes, Type-III effectors

## Abstract

**Background:**

*Salmonella enterica* subsp. *diarizonae* (IIIb) is frequently isolated from the environment, cold-blooded reptiles, sheep and humans; however only a few studies describe the isolation of this subspecies from invasive human infections. The factors contributing to this unusual behavior are currently unknown.

**Results:**

We report here the genome features of two *diarizonae* strains, SBO13 and SBO27, isolated from endocervical tissue collected post-abortion and from cerebrospinal fluid of a newborn child, respectively, in the city of Santa Cruz, Bolivia. Although isolated six years apart, SBO27 in 2008 and SBO13 in 2014, both strains belong to the same sequence type 1256 (ST1256) and show a high degree of genome conservation sharing more than 99% of their genes, including the conservation of a ~ 10 kb plasmid. A prominent feature of the two genomes is the presence of 24 genomic islands (GIs), in addition to 10 complete *Salmonella* pathogenicity islands (SPI) and fragments of SPI-7, a pathogenicity island first reported in the human-adapted serovar Typhi. Some of the GIs identified in SBO13 and SBO27 harbor genes putatively encoding auto-transporters involved in adhesion, lipopolysaccharide modifying enzymes, putative toxins, pili-related proteins, efflux pumps, and several putative membrane cation transport related-genes, among others. These two Bolivian isolates also share genes encoding the type-III secretion system effector proteins SseK2, SseK3 and SlrP with other *diarizonae* sequence types (ST) mainly-associated with infections in humans. The *sseK2*, *sseK3* and *slrP* genes were either absent or showing frameshift mutations in a significant proportion of genomes from environmental *diarizonae* isolates.

**Conclusions:**

The comparative genomic study of two *diarizonae* strains isolated in Bolivia from human patients uncovered the presence of many genes putatively related to virulence. The statistically-significant acquisition of a unique combination of these functions by *diarizonae* strains isolated from humans may have impacted the ability of these isolates to successfully infect the human host.

**Electronic supplementary material:**

The online version of this article (10.1186/s12864-018-5352-z) contains supplementary material, which is available to authorized users.

## Background

The genus *Salmonella* comprises Gram-negative pathogenic bacteria that infect a large variety of hosts, including humans, livestock, reptiles, birds and insects [1]. The genus includes two species, *S. enterica* and *S. bongori,* with *S. enterica* further subdivided in six subspecies: *enterica* (I), *salamae* (II), *arizonae* (IIIa), *diarizonae* (IIIb), *houtenae* (IV), and *indica* (VI) [1].

*S. enterica* subsp. *enterica* is responsible for most infections in warm-blooded (homeotherm) animals and comprises more than 1580 serovars that are differentiated by distinct somatic (O-antigen of the lipopolysaccharide, LPS) and flagellar antigenic formulae [2]. Some of these serovars are host-adapted while others cause diseases in a broad range of hosts [3]. Host-adapted serovars are frequently associated with systemic diseases like typhoid fever. In contrast, non-typhoidal *Salmonella* (NTS) often cause gastroenteritis in immunocompetent hosts. NTS serovars have also been linked to bacteraemia in immunocompromised and even in immunocompetent individuals [4]. The most common NTS isolated from human patients include Enteritidis, Typhimurium, Infantis and Newport [5, 6].

Although more than 99% of the *S. enterica* strains isolated from human patients belong to the subsp. *enterica*, it is not unusual, especially in children, to find reports describing isolation of strains belonging to other subspecies [7]. Many of these cases involve *Salmonella* infections linked to contact with cold-blooded animals, like reptiles kept as pets [8, 9]. Some studies have shown the presence of all *Salmonella* subspecies as part of the reptile gut microbiota, which may represent a risk of transmission to more susceptible (warm-blooded) hosts [8].

Pet snakes are reported to be an important reservoir of *S. enterica* subsp. *diarizonae* (hereafter *diarizonae*) [10]. This subspecies is also often isolated from sheep [11–14] and is increasingly associated with infections in humans. Although in some of these cases the source of the infection could be traced to reptile pets and consumption of sheep meat [15–17]; it remains unknown whether human-to-human transmissions occur.

In this study, we report the genome analysis of two *diarizonae* strains with antigenic formula 48:i:z and sequence type ST1256, which were isolated from human patients in the city of Santa Cruz, Bolivia. Although these two isolates were isolated 6 years apart (2008 and 2014), our analysis shows a high degree of genome synteny between them. Remarkably, these two Bolivian isolates share a defined set of putative virulence genes with *diarizonae* of other sequence types (ST) mainly-associated with human infections. These factors include some effector proteins translocated by type III secretion systems, a genomic island (GI-6) and a fragment of the *Salmonella* pathogenicity island 7 (SPI-7).

## Results and discussion

### Molecular typing of SBO13 and SBO27 strains

Pet snakes are common reservoirs of *diarizonae* and, consequently, potential transmission sources. However, invasive infections in humans are rarely reported [10, 15, 17, 18]. Accordingly, we were interested in analyzing the genome of two *diarizonae* strains isolated from two human samples in the city of Santa Cruz, Bolivia. These strains were cultured from cerebrospinal fluid of a 36 h-old newborn that died (strain SBO27, isolated in 2008) and, endocervical tissue collected from a 23-year old woman who miscarried after ten weeks of pregnancy (strain SBO13, isolated in 2014). There was no record of the immune status of the infected pregnant woman whereas no immune or weight defects were observed in the newborn. This newborn was however delivered by a mother having urinary infections during pregnancy.

In the initial phenotypic analyses, isolates SBO13 and SBO27 were identified as *diarizonae*, as they are positive for malonate utilization but negative for dulcitol fermentation. Plasmid profile analyses showed the presence of one plasmid of ~ 10 kb in both strains. Antimicrobial susceptibility tests indicated that SBO13 and SBO27 are susceptible to most common beta-lactams, aminoglycosides, trimethoprim-sulfametoxazol and quinonoles.

Both SBO13 and SBO27 strains were assigned to serovar IIIb 48:i:z by serological and molecular methods, according to the White-Kauffmann-Le Minor scheme [2]. Multi-locus sequence typing (MLST) analysis revealed that both isolates belong to ST1256, according to the Achtman scheme [19]. Serovar designation and ST were confirmed using the genome sequence and the ST prediction tool available in the EnteroBase database (https://enterobase.warwick.ac.uk/).

### Genome characteristics of the *diarizonae* isolates SBO13 and SBO27

The draft genomes of the *diarizonae* isolates SBO13 and SBO27 have 5,031,187 and 5,035,353 bp, respectively, with a G + C average content of 51.4%. The genome of SBO13 has a coding capacity for 4649 predicted proteins, 8 rRNAs, 80 tRNAs, and 165 ncRNAs (GenBank accession #PVNR00000000). The coding capacity of the SBO27 genome accounts for 4635 predicted proteins, 8 rRNAs, 80 tRNAs and 165 ncRNAs (GenBank accession #PVNQ00000000). SBO13 and SBO27 harbor one plasmid of 9377 and 9246 bp, respectively, with a notorious synteny and similar gene content (Additional file 1: Table S1). No antibiotic resistance-related genes were identified in these small plasmids, which encode several hypothetical proteins of unknown function, two putative regulatory proteins and an integrase (Additional file 1: Table S1).

Despite the sensitivity of SBO13 and SBO27 to many antimicrobials, a search in the ‘Comprehensive antibiotic resistance database’ (CARD) (https://card.mcmaster.ca/), revealed the presence of a reduced number of genes linked to antimicrobial resistance in both isolates. These consisted mainly of genes encoding components of efflux pumps of the major facilitator superfamily (MFS). Mutant alleles in *glpT* and *uphT*, which confer fosfomycin resistance based on a target modification mechanism, were also detected (Additional file 2: Table S2).

The genomes of SBO13 and SBO27 were initially annotated with Prokka [20] for phylogenomic analyses (see Methods) and subsequently by NCBI following our submission. To functionally analyse the SBO13 and SBO27 genomes, we used the subsystem annotation available at RAST server (http://rast.nmpdr.org/) [21, 22], which allows classification by biological processes. In SBO13, a total of 2771 protein-encoding genes were allocated to 571 annotated subsystems, defined as biological processes or structural complexes supported by a set of functional roles [20]. Most of these genes were predicted to be involved in carbohydrate (697 genes) and amino acid (439 genes) metabolism (Additional file 3: Figure S1). Interestingly, a considerable number of genes, 237, were predicted to be associated with transport-related processes and another 130 genes predicted to encode protein secretion systems, some of which have been identified as important traits involved in bacterial virulence and pathogenesis [23]. Similar results regarding the distribution of genes in the different subsystems categories were obtained for the SBO27 genome (not shown).

### Phylogenetic analysis of *diarizonae* SBO13 and SBO27 genomes

**Fig. 1:**
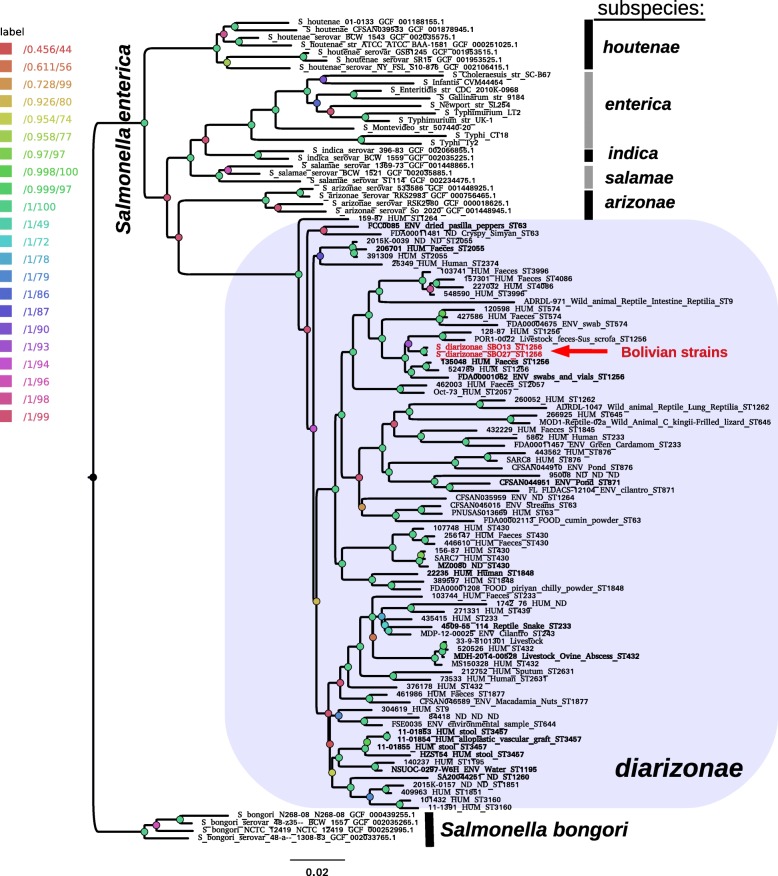
Maximum likelihood pan-genome phylogeny of the genus *Salmonella*. The tree was estimated from the presence-absence matrix of 18,446 consensus homologous gene clusters found among 109 *Salmonella* spp. genomes with GET_HOMOLOGUES. Seventy-six of them correspond to a selection of *S. enterica* subsp. *diarizonae* sequences downloaded from ENTEROBASE, thirty-one to reference genomes from other species and serovars, and the remaining two to the Bolivian strains, SBO13 and SBO27. The matrix contains 4177 distinct patterns, 9276 parsimony-informative, 6963 singleton and 2207 constant sites. Nodal support values are color-coded as shown on the legend, the first value corresponding to approximate Bayesian support values and the second one to ultra-fast bootstrap values, as implemented in IQTREE. The scale represents the number of expected substitutions per site under the best-fitting GTR2 + F0 + R2 (binary) model. The phylogeny corresponds to the top-scoring tree (ln*L* = − 197,100.501) found among 10 independent IQTREE searches. It was rooted at the *S. bongori* clade. Labels for the 76 *S. enterica* subsp. *diarizonae* genomes fetched from ENTEROBASE indicate relevant ecological metadata and the sequence type (ST). Genomes in bold were selected for further analysis

To infer the phylogenetic affinities of the SBO13 and SBO27 genomes, we estimated a maximum-likelihood phylogeny from the consensus pan-genome (presence-absence) matrix of 18,446 consensus clusters computed from 109 genomes representative of the distinct *Salmonella* species and subspecies (Fig. 1). From the 551 *diarizonae* genomes available in EnteroBase in an accessible status (Additional file 4: Table S3), we included in the analysis 70 draft (Additional file 5: Table S4) and 6 complete genomes (Additional file 6: Table S5). These were selected based on two main criteria. First, the different source and geographic location of the isolate; and, secondly, a sample size roughly proportional to the number of isolates with a particular ST (see Methods).

The pan-genome phylogeny resolved the *S. bongori* and *S. enterica* species clades and the six *S. enterica* subspecies with maximal support (Fig. 1). The Bolivian isolates SBO13 and SBO27 formed a perfectly supported tree with other ST1256 isolates. The remaining *diarizonae* genomes also grouped mainly by their respective ST (Fig. 1). However, there was no apparent clustering of the *diarizonae* genomes by their isolation source, implying that no major differences in genome content exists among *diarizonae* isolates from human, animal, plant or environmental sources (Fig. 1). This is reflected by the observation that most major *diarizonae* clades contain strains from different sources (Additional file 4: Table S3). On the other hand, the maximum-likelihood core-genome phylogeny computed from 1058 top-scoring markers selected by the GET_PHYLOMARKERS pipeline [24] out of the 1650 consensus clusters (see Methods) computed by GET_HOMOLOGUES [25], resolved the same major groups (species and subspecies) within the *Salmonella* genus; although, as expected, with much lower resolution within the *S. enterica* subspecies (Additional file 7: Figure S2).

As mentioned above, the MLST analyses showed that SBO13 and SBO27 belong to the ST1256 (Additional file 5: Table S4). This is the fourth most abundant ST for *diarizonae* found in EnteroBase as of September 2018 (https://enterobase.warwick.ac.uk/). Interestingly, *diarizonae* strains with ST1256 are recovered mainly from human infections (37 of 40 isolates) (Additional file 4: Table S3). To date, isolates of ST1256 have been only reported in Europe and North America (Additional file 4: Table S3), which make SBO13 and SBO27 the first cases of *diarizonae* ST1256 isolated from South America. Similar to ST1256, other abundant *diarizonae* STs also display association with human infections. That is the case of ST233 (37 of 52 isolates); ST430 (21 of 31 isolates); and, ST432 (15 of 51 isolates, some of them with no source information). There are, however, other abundant STs, like ST63, isolated mainly in North America, for which human isolates are rare (1 of 28 isolates) (Additional file 4: Table S3). These differences also depend on important parameters such as the geographic location in which the strains were obtained. For example, a high percentage of the *diarizonae* STs strongly associated with human infections (ST233, ST430 and ST432); however, most strains were isolated in the United Kingdom (Additional file 4: Table S3). Therefore, this apparent association between STs and human sources may in part reflect a sampling bias. Overall, *diarizonae* strains appear to have a broad ecological valence, which indicates that much remains to be learned about their environmental distribution, molecular basis of host colonization and tissue tropism.

### Genomic islands (GIs) of *diarizonae* SBO13 and SBO27 strains

**Fig. 2:**
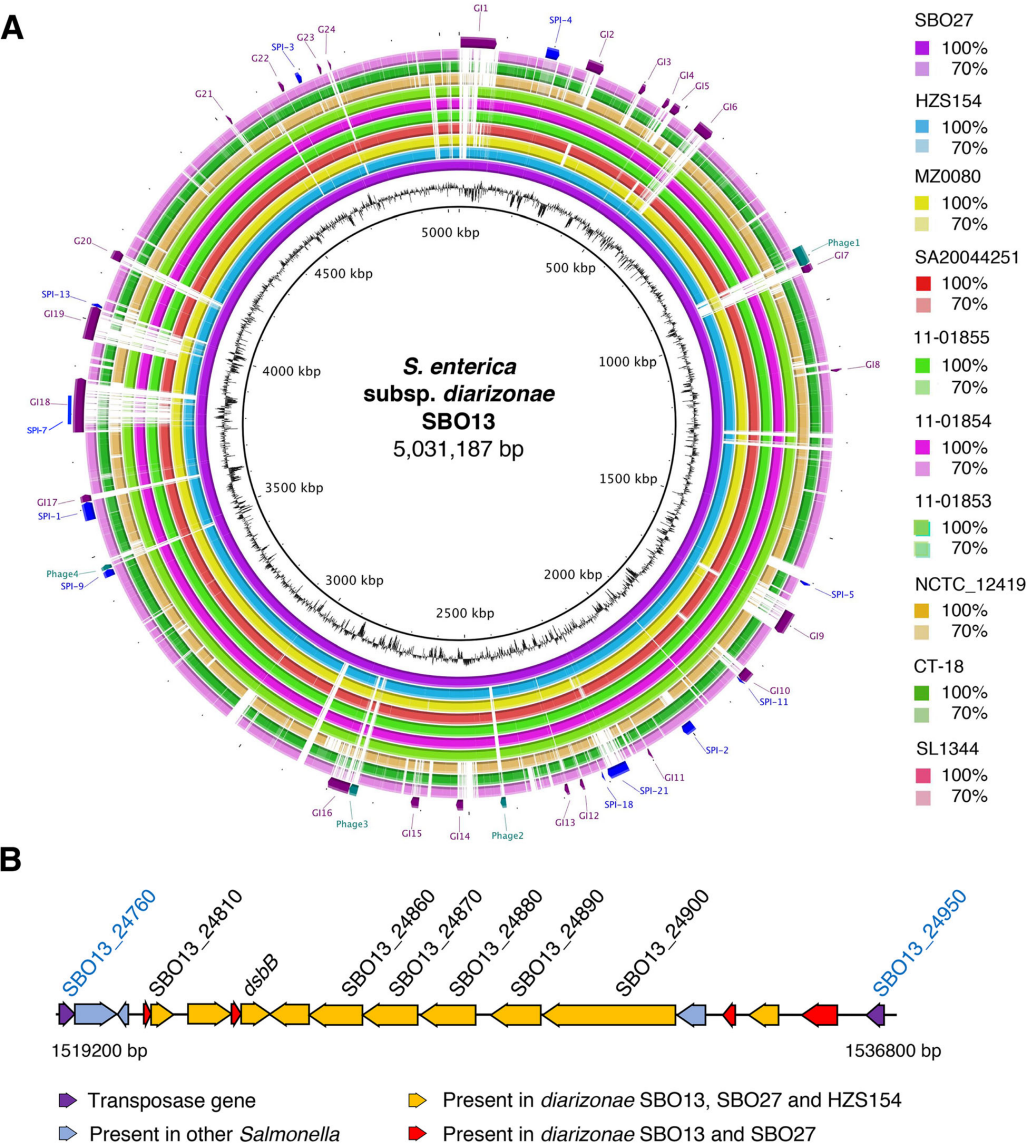
Genome comparison of *S. enterica* subsp. *diarizonae* SBO13 to nine genomes from representative *Salmonella* species, subspecies and serovars. (A) The innermost rings show *diarizonae* SBO13 genome position (kbp = kilo base pairs); G + C content (black). The remaining rings (from ring 3 to ring 12) correspond to the genomes of *diarizonae* strains SBO27, HZS154, MZ0080, SA20044251, 11–01855, 11–01854 and 11–01853; *S. bongori* NCTC-12419; Typhi CT18, and Typhimurium SL1344. BLASTN matches with an identity between 50 and 100% are colored in gradient, while nonmatching regions appeared as blank spaces in each ring. The outer ring contains genomic islands (GIs) predicted for *diarizonae* SBO13 with Islandviewer4 (http://www.pathogenomics.sfu.ca/islandviewer/). (B) Detail of a GI-6 fragment of the *diarizonae* SBO13 genome showing unique genes and genes shared exclusively with *diarizonae* HZS154, a human isolate reported in China. Highlighted with their corresponding gene numbers are the genes of this GI-6 cited in the text

To further characterize the genomes of *diarizonae* isolates SBO13 and SBO27, an all-against-all BLAST comparison was performed using nine complete genomes from distinct *Salmonella* species, subspecies, and serovars. These genomes included: six *diarizonae* strains with complete genome (Additional file 6: Table S5); *S. bongori* (strain NCTC_12,419); serovar Typhi (strain CT18); and serovar Typhimurium (strain SL1344) (Fig. 2a). A total of 24 genomic islands (GIs), designated as GI-1 to GI-24 were predicted in SBO13 and SBO27 using SIGIHMM and IslandPath-DIMOB (Fig. 2a, Additional file 8 : Table S6). Two islands, GI-17 and GI-20, were found in SBO13 and SBO27 and mostly absent in the rest of the genomes analysed (Fig. 2a). Detailed inspection of GI-17 and GI-20 revealed the presence of predicted phage-related genes along with a high proportion of genes encoding hypothetical proteins, suggesting that they are likely remnants of prophages acquired by these two strains. In addition, SBO13 and SBO27 contain an additional 11.8 kb terminal extension at GI-18 that is absent in the rest of strains analysed, making it the largest GI found in *diarizonae* (115 kb). This GI-18 region harbors genes encoding for predicted proteins related to virulence such as fimbrial proteins, an efflux system and a putative toxin (Additional file 8: Table S6). Interestingly, the analysis of two other large islands, GI-1 and GI-19, also revealed the presence of genes putatively involved in virulence. For example, genes encoding predicted proteins with homology to toxin-coregulated pilus biosynthesis-like proteins E (TcpE) (SBO13_30280) and TcpQ (SBO13_30220) [26], were found in GI-1; while several genes predicted to be related to iron transport such as the ferrous iron permease, *efeU,* the cytoplasmic ferritin, *ftn,* or the Fur regulated iron transporter, *sitA,* were identified in GI-19 (Additional file 8: Table S6). Accordingly, it is known that iron increases the pathogenic potential of *Salmonella* and other enteric pathogens during the infection of the intestinal epithelium [27].

On the other hand, some of the genes present in GI-6 and shared by SBO13 and SBO27 define a cluster of genes that is flanked by two transposase-encoding genes and is mostly predicted to be related to membrane transport (Fig. 2b, Additional file 8: Table S6). Most genes of this cluster have homologs in *diarizonae* strain HZS154 (isolated from stool of a human patient), including an auto-transporter bearing an outer membrane beta-barrel domain (SBO13_24810, SBO13_24900), a disulfide bond formation protein B, DsbB (SBO13_24840), an ABC-transporter (SBO13_24870), an iron permease (SBO13_24860), an outer membrane porin (SBO13_24880) and an O-antigen LPS length determinant protein (SBO13_24890), among others (Fig. 2b, Additional file 8: Table S6).

### Pathogenicity islands (SPIs) in *diarizonae* SBO13 and SBO27

Although there are few human clinical reports from *S. enterica* isolates not belonging to subsp. *enterica* [7, 18], *diarizonae* has been found sporadically responsible for maxillary sinusitis, bacteremia, cervical lymphadentitis and gastroenteritis in adults and children [15, 17, 28, 29]. Our whole genome comparison revealed that *diarizonae* SBO13 and SBO27 harbor 10 known *Salmonella* pathogenicity islands (SPI-1, SPI-2, SPI-3, SPI-4, SPI-5, SPI-9, SPI-11, SPI-13, SPI-18, and SPI-21) and a significant portion of SPI-7 (Fig. 2a). SPI-21, which encodes a type VI secretion system as well as the bacterial protein toxins pyocins, is shared only by *diarizonae* and *arizonae* and has been proposed to enable these two subspecies to compete with other bacteria [30]. Of interest, all *diarizonae* genomes lack SPI-6 and SPI-12, which have been shown to be important for competition and virulence in subsp. *enterica* [31, 32].

**Fig. 3:**
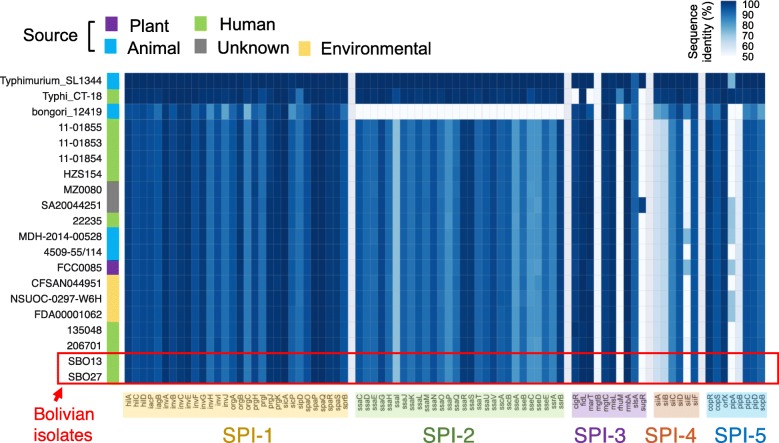
Heatmap of the SPI1-SPI5 regions in the *S. enterica* subsp. *diarizonae* genomes. TBLASTN searches, using the protein sequences from serovar Typhimurium SL1344 strain as query, were done to identify orthologous genes in *S. bongori* NCTC-12419, serovar Typhi CT-18, and 15 representative *diarizonae* genomes. Orthologous genes with 85% sequence coverage were considered present, and marked in blue. The color intensity represents the sequence identity from 50 to 100% (complete protein). Orthologous genes that did not fulfil the criteria outlined above, were considered absent, and marked in white. Phage remnants regions, SBO13 genomic islands (GI) and *Salmonella* pathogenic islands (SPI) are indicated in cyan, purple and dark blue, respectively

The most intensively studied pathogenicity islands in *Salmonella* are SPI-1, SPI-2, SPI-3, SPI-4, and SPI-5, which play important role in adhesion, invasion and the intracellular lifestyle of the pathogen [33]. To assess putative differences in these five islands among *diarizonae* strains, we analysed their gene content by BLAST in SBO13 and SBO27 and, in a representative group of 15 *diarizonae* strains collected from human, animal, plant and environmental sources (Additional file 6: Table S5). Among these are included those six *diarizonae* isolates with known complete genome used in previous analyses. High conservation in gene content was observed for these pathogenicity islands (SPI-1 to SPI-5) in all *diarizonae* isolates, irrespective of their origin (Fig. 3).

SPI-7 in serovar Typhi comprises a DNA region of 134 kb, which contains approximately 150 predicted genes [34]. In addition, SPI-7 has a mosaic structure, comprising regions thought to be involved in island mobility and other regions implicated in virulence: the *viaB*- operon, related to production of Vi antigen; a prophage region encoding the SopE type-III effector; a type-IV secretion system (*pil-locus*); and, the *rep*-cluster, with homology to a plasmid replication regions [35]. SPI-7 was first discovered in Typhi but it has been also found in serovar Paratyphi C and some strains of serovar Dublin [36, 37]. Noteworthy, although a recent study showed SPI-7 to be present in the genome of *diarizonae* strain 1579 and absent in strain 639, no further details about the gene content of this particular SPI-7 fragment were provided [31].

To gain further insights into the organization of the *diarizonae* genomic region containing SPI-7 genes, a sequence homology analysis using SPI-7 genes from Typhi strain CT-18 was run with the same set of *diarizonae* genomes used in the analysis of SPI-1 to SPI-5 islands: 15 representative *diarizonae* strains from diverse sources along with SBO13 and SBO27 (Additional file 6: Table S5). The initial TBLASTN analysis using as queries SPI-7 proteins from Typhi strain CT-18 detected 66 of a total of 128 genes of the SPI-7 island in SBO13 and SBO27 with a coverage of ≥85%. Moreover, the SPI-7 genes found showed a high synteny compared to Typhi SPI-7 (Additional file 9: Figure S3, see below). Since a high proportion (22/66) of those TBLASTN-hits showed low-medium identity at the protein level (~ 40–50%), we decided to assess the TBLASTN analysis using an identity cut-off > 40%.

**Fig. 4:**
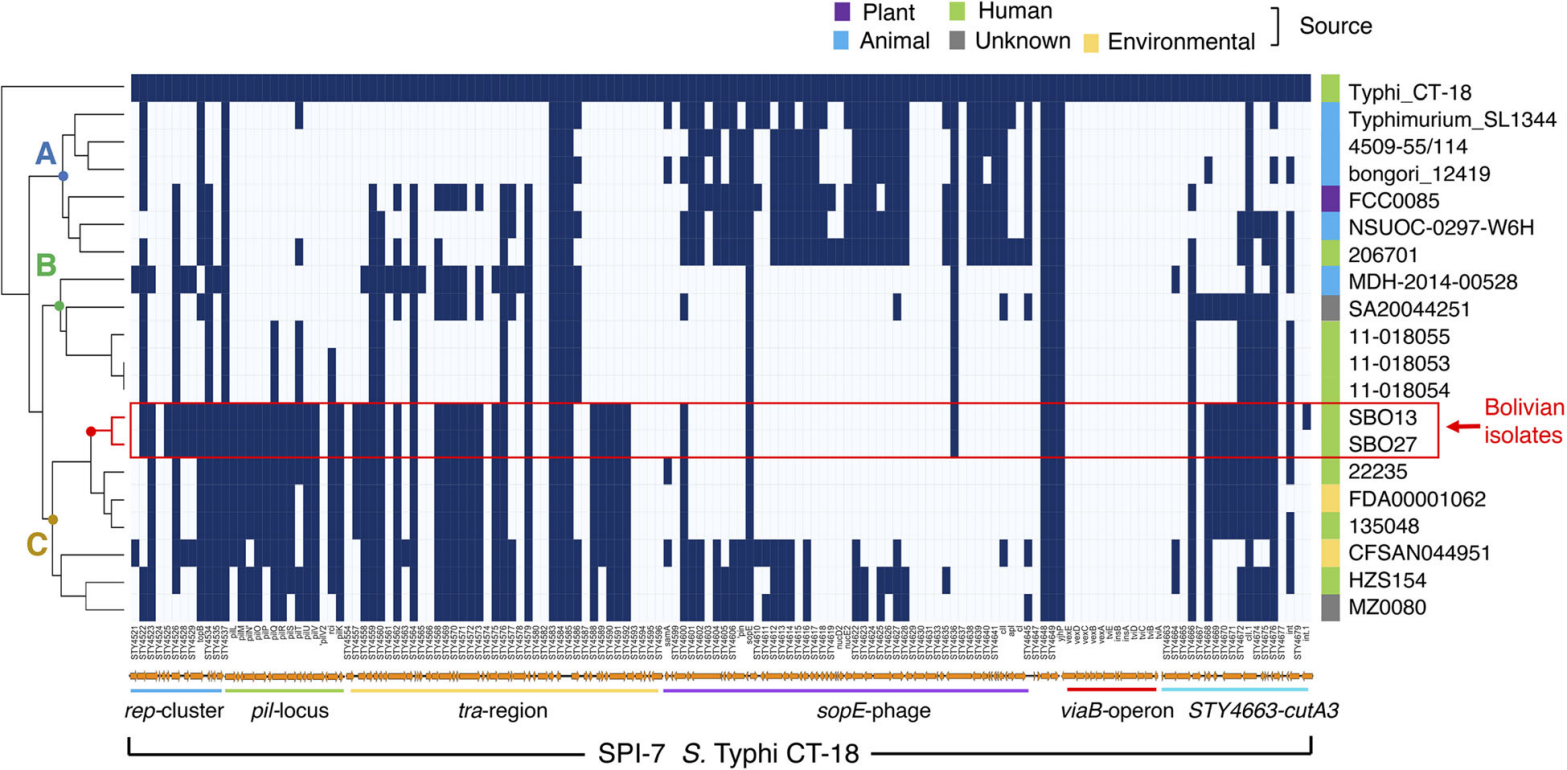
Clustering map of the SPI-7 region in representative *S. enterica* subsp. *diarizonae* genomes. TBLASTN searches, using the protein sequences from serovar Typhi strain CT-18 as query, were done to identify orthologous genes in *S. bongori* NCTC-12419, serovar Typhimurium SL1344 and 15 representative *diarizonae* genomes. Orthologous genes with 85% sequence coverage and 40% sequence identity were considered present and marked in blue. Orthologous genes that did not fulfil the criteria outlined above, were considered absent, and marked in white. The SPI-7 island of Typhi CT-18 strain and its different regions are depicted below of the clustering map

The SPI-7 gene distribution divided the 17 *diarizonae* strains analyzed in three main clades, A, B and C (Fig. 4). Clade A, which bears a significant portion of the *sopE* phage but lacking other virulence-related regions, included strains outside the subsp. *diarizonae* and a few *diarizonae* strains from diverse sources (Fig. 4). Clade B, composed of *diarizonae* strains from diverse sources, missed a large portion of SPI-7 while clade C, in which SBO13 and SBO27 were present, bear a significance portion of SPI-7 genes predicted to be related to virulence (*pil*-locus and *tra*-region) (Fig. 4). As expected, the Vi capsule polysaccharide-biosynthetic operon (*viaB*) was absent in all the *diarizonae* strains analysed (Fig. 4).

In the case of the Bolivian isolates SBO13 and SBO27, we found homology with several regions of serovar Typhi SPI-7: the *rep* cluster (homology to plasmid replication region), the *pil* locus, genes from the *tra* region involved in DNA transfer, and most genes from the *STY4663-cutA3* region, including an ortholog of the Typhi integrase *int-4680* (Fig. 4 and Additional file 9: Figure S3). The only region implicated in virulence found in this *diarizonae* SPI-7 fragment was therefore the type IVB pilus (*pil*) locus (Fig. 4). The type-IVB pilus system has been characterized in Typhi and Dublin as an important element in adhesion to and/or invasion of human epithelial cells [36, 38]. This type IVB pilus is also involved in increasing the inflammatory response in monocytes [39]. Hence, the presence of the *pil* locus in *diarizonae* SBO13 and SBO27 could have contributed to their ability to infect humans. For some *pil* gene products, the level of amino acid identity of the SBO13 and SBO27 pili proteins was ≤50% when compared with the orthologous Typhi pili proteins. This observation opens the possibility of different acquisition events of the locus, at least for some sub-regions (Additional file 9: Figure S3). Interestingly, despite showing high sequence identity between *diarizonae* and Typhi, the *STY4663*-*cutA3* cluster appears upstream of *parB-ssb* region in *diarizonae* instead of the 3′ terminal region as in Typhi (Additional file 9: Figure S3). It is also notorious the lack of genes coding for the Vi capsule polysaccharide (*viaB* operon) and, as mentioned above, the absence of most of the SopE-phage. This scenario is reminiscent of that seen in other pathogens harboring genomic islands that share synteny with SPI-7 such as *Pseudomonas fluorescens, Ralstonia metallidurans* or *Yersinia enterocolitica* [35]. In all these cases, the SPI-7 *viaB* operon is either absent or replaced by other genes.

### Phage analyses

Bacteriophages and phage remnants are found in most of *Salmonella* genomes and play an important role in both inter-species and inter-strain variability [40, 41]. Four regions were identified in the chromosome of *diarizonae* SBO13 and SBO27 using the PHASTER tool (Fig. 2a, Additional file 10 : Table S7). PHASTER predicted the presence in these four regions of ‘incomplete’ set of genes, therefore likely corresponding to phage remnants. Two of these phage remnants -numbers 3, 4 in SBO13; and, 2, 3 in SBO27- have the same length and G + C content in the two strains, suggesting that they correspond to the same genetic element. The identified phage remnants displayed a mosaic of genes from related bacteriophages such as SEN34, Gifsy1, P4, and prophage elements from other bacterial genera. Among the genes mapping to these phage remnants, we found one encoding the virulence factor PagK. This protein and its homologues (PagJ and PagK2) belong to a set of virulence factors proposed to be translocated into the host cytoplasm of macrophages via bacterial outer membrane vesicles [42]. It has been reported that *Salmonella* strains lacking these three homologues are attenuated for virulence in murine infections [36] (Additional file 10: Table S7). The precise role of these phage remnants, which encode a high number of hypothetical proteins, in the biology of SBO13 and SBO27 is yet to be explored.

### Virulence factors in *diarizonae* SBO13 and SBO27

Effector proteins translocated into eukaryotic host cells by specialized type-III secretion systems (T3SS) contribute largely to *Salmonella* virulence [43–45]. The combined action of these effector proteins allows *Salmonella* to alter host cell functions enabling intracellular survival and replication within eukaryotic cells and transmission [44, 45]. Likewise, many studies have demonstrated a prominent role of diverse adhesins in promoting the interaction of *Salmonella* with host cells [46].

**Fig. 5:**
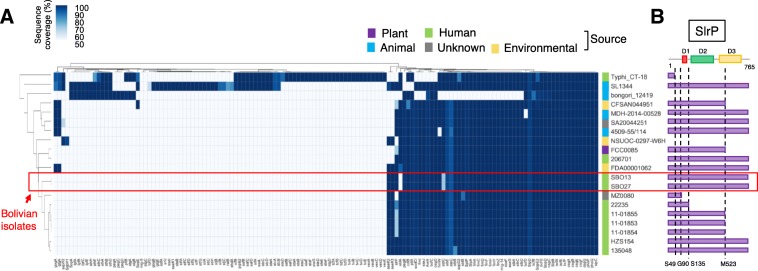
Distribution of virulence-related functions in representative *S. enterica* subsp. *diarizonae* strains, including SBO13 and SBO27. **a** TBLASTN searches using as queries all the virulence factors available from VFDB (http://www.mgc.ac.cn/VFs/) excluding SPI1, SPI2 and those presents in plasmids. Proteins with 70% sequence identity were considered present and marked in blue. The color intensity represents the sequence coverage from 50 to 100% (complete protein). Proteins that did not fulfil the criteria outlined above, were considered absent, and marked in white. **b** Schematic representation of SlrP sequences found are represented as purple bars on the right panel. The protein domains of SlrP are represented as coloured boxes: type III secretion system leucine rich repeat region (TTSSLRR; D1 in red), Leucine rich repeat region (LRR; D2 in green) and C-terminal novel E3 ligase domain (NEL; D3 in yellow)

To further investigate virulence-related traits in *diarizonae* SBO13 and SBO27, we searched by TBLASTN the presence of type-III effector proteins and adhesin operons in the Virulence Factors DataBase (VFDB, http://www.mgc.ac.cn/VFs/) (Additional file 11: Table S8) using the set 15 *diarizonae* genomes previously described (Additional file 6: Table S5). This comparative analysis revealed that 12 out of 41 type III effectors were encoded by all the *diarizonae* genomes: SteC, SseJ, SseG, SseF, SptP, SopE, SopE2, SopB, SipA, SipB, SipD and AvrA (Fig. 5). Interestingly, 11 out of the 17 *diarizonae* strains examined, including SBO13 and SBO27, harbor genes encoding the glycosyltransferases SseK2 and SseK3, which impair proper immune response to infection via TNFα-stimulated NF-κB signaling [43]. Noteworthy, 8 out of the 9 *diarizonae* strains associated with human infections were positive for *sseK2* and *sseK3* (Fig. 5). Moreover, an extra type III effector, SlrP, was found in some of the strains associated with human and animal infections (Fig. 5). SlrP has E3 ubiquitin ligase activity and has been shown to inhibit the release of IL-1β [43]. A detailed inspection of the SlrP amino acid sequence across the *diarizonae* strains analyzed revealed that SlrP was present in some isolates as a truncated protein due to a frame-shift mutation (Fig. 5). As mentioned above, SrlP appears as the full-length version in the Bolivian strains SBO13 and SBO27 (Fig. 5). SlrP interacts with mammalian thioredoxin-1 leading to a decrease in thioredoxin activity and host cell death [47]. Hence, the incorporation of SlrP together with the glycosyltransferases SseK2 and SseK3 might have facilitated infection in humans by the SBO13 and SBO27 isolates. Noteworthy, no selective acquisition or loss of fimbrial operons was observed for the *diarizonae* isolates examined. The only exception was the *stdA* and *stdC* fimbrial genes, which in the Bolivian isolates SBO13 and SBO27 encode proteins with a low coverage (lower than 50%) in comparison to other *diarizonae* strains (Fig. 5).

### Presence of SBO13 and SBO27 virulence determinants in *diarizonae* draft genome assemblies available at EnteroBase

The genome analysis of *diarizonae* SBO13 and SBO27 revealed a defined set of distinctive features linked to virulence such as the SPI-7 *pil-locus*, the transport-related cluster of GI-6, and genes the T3SS effectors SlrP, SseK2 and SseK3. To get additional insights about the distribution of this set of genes in other *diarizonae* strains, the presence of these markers was assessed by TBLASTN in the 376 *diarizonae* draft genome assemblies available at EnteroBase (https://enterobase.warwick.ac.uk/) (Additional file 12: Table S9 and Additional file 13: Table S10). These 376 genomes comprised those ST with a minimum of 5 isolates. The SPI-7-related *pil*-locus was found in 44.8% of the *diarizonae* genomes whereas the GI-6 cluster was found only in 29.6% of them (Additional file 14: Table S11). Therefore, these genome regions are present in less than half of the *diarizonae* genomes analyzed. The genes encoding the T3SS effectors SlrP, SseK2 and SseK3 were found in 80.5, 46.9 and 45.9% of the 376 *diarizonae* genomes, respectively (query coverage ≥85%; Additional file 14: Table S11). To test the possibility that these virulence factors -SlrP, SseK2, SseK3, GI-6 and SPI-7- could be involved in facilitating human infection, we analyzed their distribution in *diarizonae* STs statistically associated with humans (Fisher exact test *p*-value < 0.05) (Additional file 13: Table S10). Among these virulence-related functions, SlrP, GI-6 and SPI-7 were not only over-represented in STs statistically associated with the human host, but also in isolates collected from human infections regardless of whether their respective STs were associated or not to human host (Additional file 14: Table S11). Even more, in five STs: 1845, 3996, 4086, 876 and 1256 (SBO13 and SBO27), recovered mainly-from human infections (Fisher exact test *p*-value < 0.05) (Additional file: 13: Table S10), these virulence determinants were presents at least in the ≥75% of the isolates (Additional file 13: Table S10). Altogether, these data suggest that this repertoire of virulence factors could contribute in subspecies *diarizonae* to increase the potential to cause infection in humans.

## Conclusions

This study provides insights into the genome structure of two *diarizonae* strains, SBO13 and SBO27, which were isolated in Bolivia from invasive infections in humans. Pan-genome phylogeny clustered these two Bolivian isolates with *diarizonae* strains of same genotype, ST1256, with no obvious differences with other *diarizonae* ST comprising mainly-human, animal or environmental isolates. However, the subsequent study of virulence-related functions encoded by the SBO13 and SBO27 genomes pointed to a unique gene repertoire that may facilitate infection of the human host. The statistical analyses revealed that, as it occurs in SBO13 and SBO27 strains, a higher percentage of *diarizonae* human isolates harbor genes encoding the type III effectors SseK2, SseK3 and SlrP; the SPI-7 *pil*-*locus,* and predicted membrane transport-related genes mapping in GI-6.

## Methods

### Isolation and characterization of *S. enterica* subsp*. diarizonae* strains SBO13 and SBO27

The strains were isolated from two human infection cases and sent to the National Centre for Tropical Diseases (CENETROP, Santa Cruz, Bolivia). Strain SBO27 was obtained from cerebrospinal fluid from a 36 h-old neonate who died in 2008, and strain SBO13 was isolated from endocervical tissue collected post-abortion at ten-weeks of gestation from a 23-year old pregnant female in 2014. The isolates were identified as *S. enterica* by standard biochemical tests. The antigenic formula was evaluated by serology and by PCR, and plasmid profiles were visualized as previously described [48]. Antimicrobial susceptibility tests were performed by the Kirby-Bauer disk diffusion method and the breakpoints were considered according to the Clinical Laboratory Standards Institute [49].

For strains SBO13 and SBO27 strains, the initial multi-locus sequence typing (MLST) was performed by PCR and sequencing as described elsewhere [19], and confirmed after genome analysis using a web tool available at https://cge.cbs.dtu.dk/services/MLST/.

### Genome sequencing and annotation

Purified genomic DNA was commercially sequenced at the Genomics Core of Arizona State University on an Illumina MiSeq instrument (~ 550 bp PE libraries; 2 × 300 sequencing cycles). Genome assembly and annotation were performed as previously described [50]. Briefly, the reads were processed with trimmomatic [51] to remove adaptors and trim poor-quality bases before de-novo assembly using spades 3.11.0 [52]. Scaffolds were ordered using multiple complete *diarizonae* genomes as references with the aid of MeDuSa v1.6 [53]. The resulting assemblies were subjected to a final polishing step by remapping reads with BWA (Li and Durbin, 2009) and using Pilon v1.22 [54] to correct the sequences and gap filling/closure. In-house annotation was performed with Prokka [20] and a local *S. enterica*-specific database of selected reference sequences (Prokka annotation data are public and available at https://figshare.com/s/38a6ebb54e78e5885b31). Sequences released to the public domain were annotated with the NCBI prokaryotic genome automatic pipeline during the submission process. *S. enterica* subsp. *diarizonae* genome assemblies downloaded from EnteroBase as FASTA-formatted contigs were likewise annotated with Prokka.

### Comparative genome analyses

The genomes were aligned with MAUVE aligner version 2.4.0 using the progressive algorithm with default settings [55]. Genome comparison was undertaken using BLAST Ring Image Generator (BRIG) [56]. *Salmonella* pathogenicity islands (SPIs), T3SS effectors and GI-6 transport related gene cluster were analyzed using TBLASTN. Putative island prediction was conducted using IslandViewer4 [57]. The genomic context of SPI-7 genes was drawn and analysed using EASYfig software [58]. Prophages sequences were predicted using the PHASTER server, a new version of PHAST [59]. Antibiotic resistance genes were searched using the Comprehensive Antibiotic Resistance Database (https://ardb.cbcb.umd.edu/blast/) [60] and Resfinder [61]. The annotation of clusters of orthologous groups (COGs) was generated using eggNOGmapper [62]. For Cluster analysis and visualization, we used the Python visualization library, Seaborn [63]. Clustering analysis was performed using the complete linkage method.

### Phylogenomic analyses

Core- and pan-genome phylogenies were estimated under the maximum-likelihood (ML) optimality criterion using the GET_HOMOLOGUES [25] and GET_PHYLOMARKERS [24] software suites. Briefly, get_homologues.pl was used in combination with compare_clusters.pl to compute a consensus core-genome, resulting from the clustering of the all-against-all BLASTP results (with query coverage ≥90%; -C 90) with the BDBH, COGtriangles and OMCL algorithms implemented in GET_HOMOLOGUES, as detailed elsewhere [64]. A consensus pan-genome was computed in a similar manner from the COGtriangles and OMCL clusters. The consensus core-genome clusters were then fed into the GET_PHYLOMARKERS pipeline to select alignments with optimal phylogenetic attributes, namely those without significant evidence for recombination, producing tree topologies and branch-lengths not significantly deviating from the expected distribution of these parameters under the multispecies coalescent, and displaying average branch support values > 0.6 (see [24] for the details). The alignments passing these filters were concatenated and a ML phylogeny estimated with IQ-TREE 1.6.1 [65] using the best fitting model and selecting the phylogeny with the highest likelihood score from those found among independent searches. The ML pan-genome phylogeny was estimated from the pan-genome (presence-absence) matrix computed by compare_clusters.pl from the GET_HOMOLOGUES suite with the aid of the estimate_pangenome_phylogenies.sh script from the GET_PHYLOMARKERS package, which calls IQ-TREE to fit the binary model to the data, and performing ten independent searches under the best-fit model. Phylogenetic trees were displayed and edited with FigTree v1.4.3.

### Comparative analyses in EnteroBase

The EnteroBase database (http://enterobase.warwick.ac.uk/species/index/senterica) was examined to determine the sequence type (STs) assignment for the strains (besides SBO13 and SBO27) included in this study and to analyze draft genomes of other *diarizonae* strains.

As of September 19, 2018; 757 *diarizonae* entries were found in EnteroBase. From these, 551 draft genome assemblies were available and downloaded as FASTA-formatted contigs. For the phylogenetic analysis, a sample of 76 *diarizonae* genomes were selected (Additional file 4: Table S3 and Additional file 5: Table S4) based on two main criteria. First, different source and geographic location of the isolation, and secondly, a representative sample roughly poportional to the number of isolates with a particular ST. We only considered those *diarizonae* genomes with STs represented in EnteroBase by more than 5 isolates. These STs were further differentiated in three classes, having 5–10, 11–20 and > 21 isolates. We further selected 2, 3 and 5 representative strains for each ST falling in each of these three classes, which conformed the 76 *diarizonae* genomes indicated above (Additional file 4: Table S3 and Additional file 5: Table S4). A subsample of 15 *diarizonae* strains was selected from the pan-genomic phylogenetic tree (Fig. 1 and Additional file 6: Table S5) and used for gene content comparative analyses (Figs. 3, 4 and 5).

For statistical association analyses, only STs with ≥5 isolates were considered from the sample of 376 genomes. These draft assemblies (FASTA contigs) were formatted with *makeblastdb*. The local genome databases were searched for the presence of the serovar Typhimurium strain SL1344 genes *slrP*, s*seK2* and *sseK3* using TBLASTN. Homologous proteins encoded by genes mapping in SPI-7 and GI-6 from strain SBO13 were searched with TBLASTN. Only hits with ≥90% query coverage and ≥ 70% of identity were considered as *bonafide* homologues (except for SPI-7, where ≥40% identity was used as cut-off for *pil-locus* genes). Fisher exact test was used to identify STs statistically associated with human host (*p*-value < 0.05).

## Additional files

Additional file 1: **Table S1.** Genes present in the plasmids of *S. enterica* subsp. *diarizonae* strains SBO13 and SBO27. (XLSK 11 kb)

Additional file 2: **Table S2.** Genes related to antibiotic resistance found in *S. enterica* subsp. diarizonae strains SBO13 and SBO27. (XLXS 18 kb)

Additional file 3: **Figure S1.** Subsystem category distribution of the *S. diarizonae* strain SBO13 genome. The genome of *diarizonae* SBO13 was annotated using the Rapid Annotation System technology (RAST) server and classified in 27 categories and 571 subsystems. Total proteins annotated with a putative function covered a 56% of the subsystems (green bar). The pie chart represents the percentage distribution of the subsystems categories. (PDF 377 kb)

Additional file 4: **Table S3.** *S. enterica* subsp. *diarizonae* genomes available in Enterobase in a accesible status (XLSX 162 kb)

Additional file 5: **Table S4.** *S. enterica* subsp. diarizonae draft genomes used in the phylogenomic analyses (XLSX 30 kb)

Additional file 6: **Table S5.** *S. enterica* subsp. *diarizonae* genomes of strains isolated from diverse sources that were further analysed in this study. (XLSX 12 kb)

Additional file 7: **Figure S2**. Maximum likelihood core-genome phylogeny (species tree) for 76 *Salmonella* spp. strains. The core-genome was inferred from the 303 top-scoring markers selected of the 1650 consensus clusters computed by GET_HOMOLOGUES. The bar represents the expected number of substitutions per site under the best-fitting GTR + F + ASC + R3 model. Internal nodes are colored by the combined approximate Bayesian support / ultra-fast bootstrap support values, respectively, as indicated on the legend. The inset shows the distribution of Robinson-Foulds gene tree distances to the species-tree. (PDF 1150 kb)

Additional file 8: **Table S6.** Genomic islands (GI) found in *S. enterica* subsp. *diarizonae* SBO13 (XLSX 43 kb)

Additional file 9: **Figure S3**. Synteny analysis of the SPI-7 island between Typhi CT18 and *S. diarizonae* SBO13 and SBO27. (A) Comparison of the entire island; (B) detail of the *rep*, *pil* and *tra* loci. The vertical grey bars represent BLAST identity of homologous regions (minimal identity for matches 40%). The gradient of the grey colour bars represents BLAST identity (%). (PDF 434 kb)

Additional file 10: **Table S7.** Phophages carried by the *S. enterica* subsp. *diarizonae* SBO13 and SBO27 (XLSX 10 kb)

Additional file 11: **Table S8.** Virulence factors proteins encoded by *S. enterica* subsp. *diarizonae* genomes and representative genomes from other *Salmonella* species and subspecies (XLSX 19 kb)

Additional file 12: **Table S9.** Presence/absence of *slrP, sseK2, sseK3*, GI6 and SPI-7 in *S. enterica* subsp. *diarizonae* draft genomes available at EnteroBase. (XLSX 39 kb)

Additional file 13: **Table S10.** *S. enterica* subsp. *diarizonae* isolates statistically associated with human infections (% versus total). (XLSX 13 kb)

Additional file 14: **Table S11.** Virulence determinants distribution along *S. enterica* subsp. *diarizonae* ST associated and non-associated with human host (% vs total). (XLSX 10 kb)

## Abbreviations

COG: Cluster of orthologous group
GI: Genomic island
ML: Maximum-likelihood
MLST: Multi-locus sequence typing
SPI: *Salmonella* pathogenicity island
ST: Sequence type
T3SS: Type-III secretion system
