# Correction to: Genome analysis of *Salmonella enterica subsp. diarizonae* isolates from invasive human infections reveals enrichment of virulence-related functions in lineage ST1256

**DOI:** 10.1186/s12864-020-06781-x

**Published:** 2020-05-26

**Authors:** Joaquín Giner-Lamia, Pablo Vinuesa, Laura Betancor, Claudia Silva, Julieta Bisio, Lorena Soleto, José A. Chabalgoity, José Luis Puente, The Salmonella CYTED Network, Fernando C. Soncini, Eleonora García-Vescovi, Griselda Flores, José Pedraza, Lucia Yim, Coralith García, Lizeth Astocondor, Theresa Ochoa, Noemí Hinostroza, M. Graciela Pucciarelli, Alfredo Hernández-Alvarez, Victor del Moral, Francisco García-del Portillo

**Affiliations:** 1 Laboratorio de Patógenos Bacterianos Intracelulares, Centro Nacional de Biotecnología-Consejo Superior de Investigaciones Científicas (CNB-CSIC), Madrid, Spain; 2 Centro de Biotecnología y Genómica de Plantas (CBGP), Universidad Politécnica de Madrid (UPM), Madrid, Spain; 3 Centro de Ciencias Genómicas, Universidad Nacional Autónoma de México, Cuernavaca, Morelos Mexico; 4 Facultad de Medicina, Instituto de Higiene, Universidad de la República, Montevideo, Uruguay; 5 Departamento de Microbiología Molecular, Instituto de Biotecnología, Universidad Nacional Autónoma de México, Cuernavaca, Morelos Mexico; 6 Ministerio de Salud de Bolivia, Centro Nacional de Enfermedades Tropicales (CENETROP), Santa Cruz, Bolivia; 7 Universidad Autónoma Gabriel René Moreno, Santa Cruz, Bolivia

**Correction to: BMC Genomics (2019) 20:99**

**https://doi.org/10.1186/s12864-018-5352-z**

Following publication of the original article [1], the authors identified an error in the processing of the name of one of the members of The Salmonella CYTED Network.

M. Graciela Pucciarelli’s middle name, Graciela, was erroneously processed as part of her Family name.
