# Supplementary material for: Genome analysis of Salmonella enterica subsp. diarizonae isolates from invasive human infections reveals enrichment of virulence-related functions in lineage ST1256
Source: BMC Genomics. 2019 Jan 31;20:99. doi: 10.1186/s12864-018-5352-z (PMC6357384; doi:10.1186/s12864-018-5352-z)
Supplement: Supplementary file 3 — Figure S1. Subsystem category distribution of the S. diarizonae strain SBO13 genome. The genome of diarizonae SBO13 was annotated using the Rapid Annotation System technology (RAST) server and classified in 27 categories and 571 subsystems. Total proteins annotated with a putative function covered a 56% of the subsystems (green bar). The pie chart represents the percentage distribution of the subsystems categories. (PDF 377 kb) [file 12864_2018_5352_MOESM3_ESM.pdf]

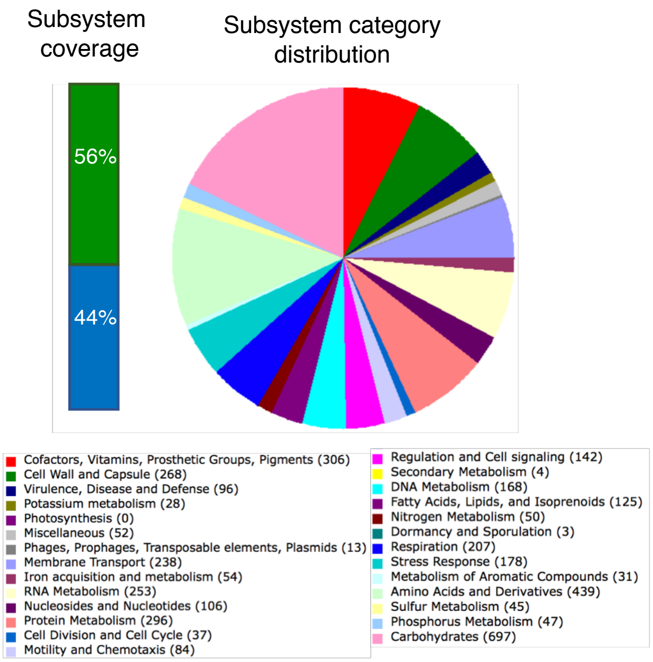

Subsystem coverage
Subsystem category distribution
56%
44%
Cofactors, Vitamins, Prosthetic Groups, Pigments (306)
Cell Wall and Capsule (268)
Virulence, Disease and Defense (96)
Potassium metabolism (28)
Photosynthesis (0)
Miscellaneous (52)
Phages, Prophages, Transposable elements, Plasmids (13)
Membrane Transport (238)
Iron acquisition and metabolism (54)
RNA Metabolism (253)
Nucleosides and Nucleotides (106)
Protein Metabolism (296)
Cell Division and Cell Cycle (37)
Motility and Chemotaxis (84)
Regulation and Cell signaling (142)
Secondary Metabolism (4)
DNA Metabolism (168)
Fatty Acids, Lipids, and Isoprenoids (125)
Nitrogen Metabolism (50)
Dormancy and Sporulation (3)
Respiration (207)
Stress Response (178)
Metabolism of Aromatic Compounds (31)
Amino Acids and Derivatives (439)
Sulfur Metabolism (45)
Phosphorus Metabolism (47)
Carbohydrates (697)
