# Supplementary figures and images for: Genome analysis of Salmonella enterica subsp. diarizonae isolates from invasive human infections reveals enrichment of virulence-related functions in lineage ST1256
Source: BMC Genomics. 2019 Jan 31;20:99. doi: 10.1186/s12864-018-5352-z (PMC6357384; doi:10.1186/s12864-018-5352-z)

*S. enterica*

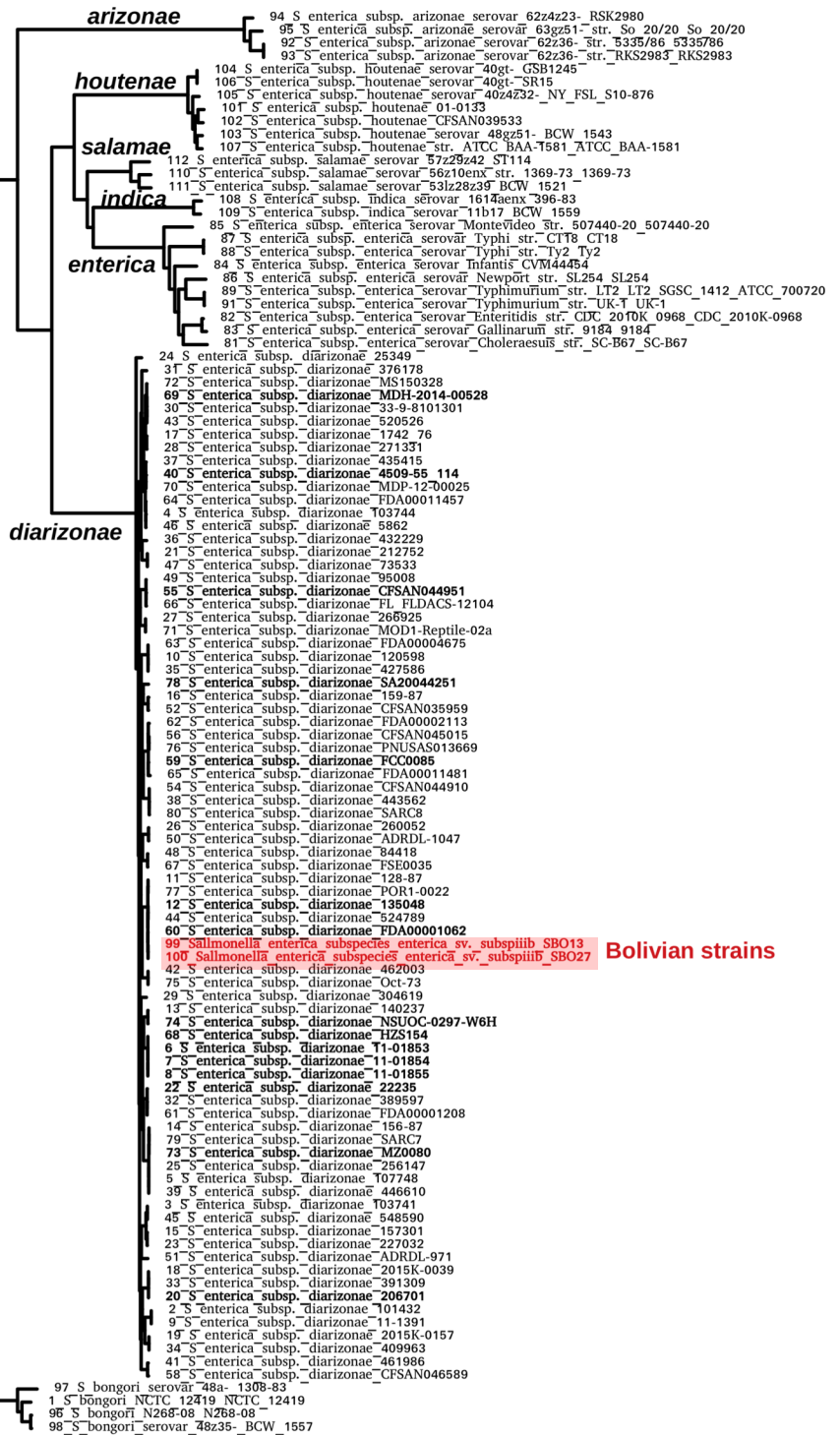

Bolivian strains

*S. bongori*

0.08

Supplement: Supplementary file 7 — Figure S2. Maximum likelihood core-genome phylogeny (species tree) for 76 Salmonella spp. strains. The core-genome was inferred from the 303 top-scoring markers selected of the 1650 consensus clusters computed by GET_HOMOLOGUES. The bar represents the expected number of substitutions per site under the best-fitting GTR + F + ASC + R3 model. Internal nodes are colored by the combined approximate Bayesian support / ultra-fast bootstrap support values, respectively, as indicated on the legend. The inset shows the distribution of Robinson-Foulds gene tree distances to the species-tree. (PDF 1150 kb) [file 12864_2018_5352_MOESM7_ESM.pdf]

Genomic island SPI7

A

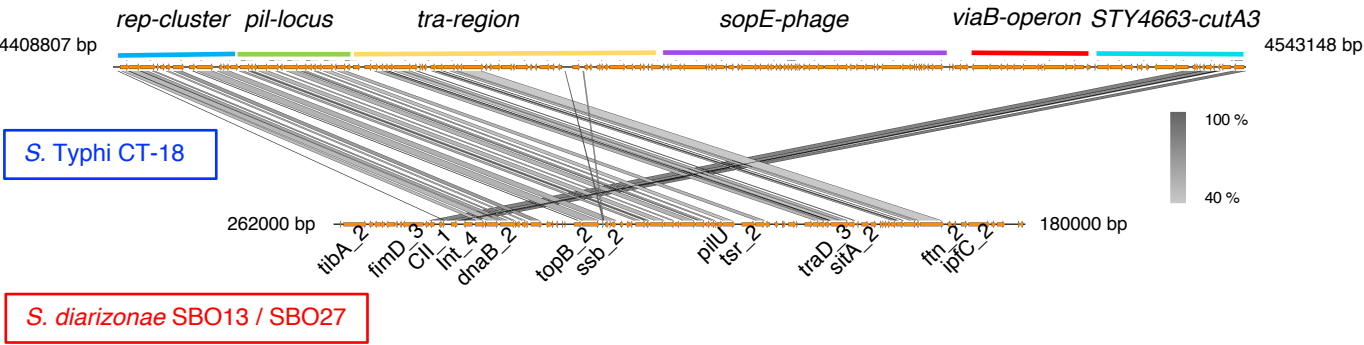

B

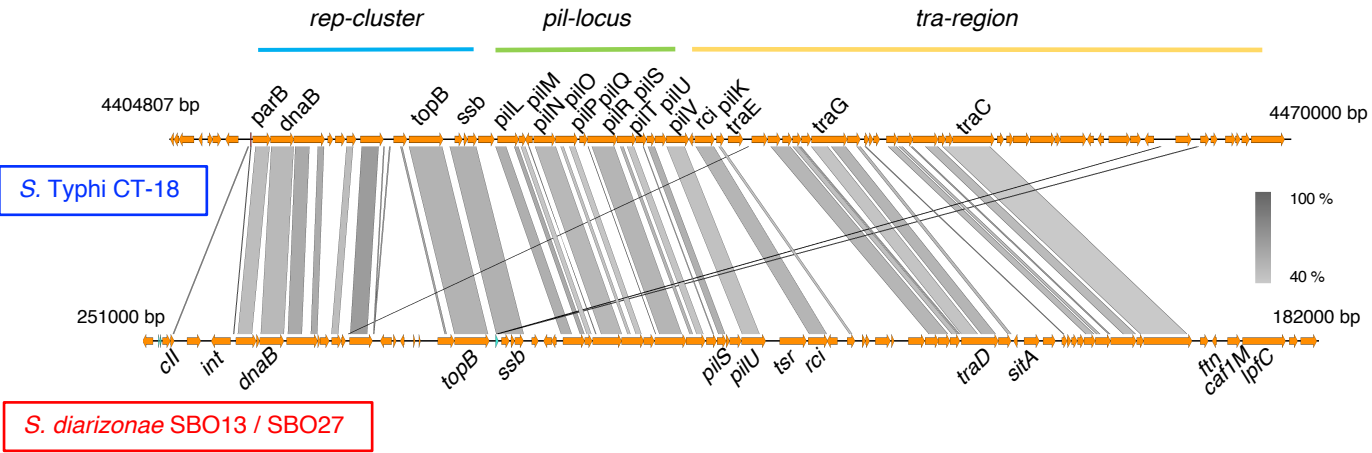

Supplement: Supplementary file 9 — Figure S3. Synteny analysis of the SPI-7 island between Typhi CT18 and S. diarizonae SBO13 and SBO27. (A) Comparison of the entire island; (B) detail of the rep, pil and tra loci. The vertical grey bars represent BLAST identity of homologous regions (minimal identity for matches 40%). The gradient of the grey colour bars represents BLAST identity (%). (PDF 434 kb) [file 12864_2018_5352_MOESM9_ESM.pdf]
